# Fully automatic quantification of pulmonary fat attenuation volume by CT: an exploratory pilot study

**DOI:** 10.1186/s41747-024-00536-z

**Published:** 2024-12-05

**Authors:** Luca Salhöfer, Mathias Holtkamp, Francesco Bonella, Lale Umutlu, Johannes Wienker, Dirk Westhölter, Matthias Welsner, Christian Taube, Kaid Darwiche, Judith Kohnke, Jannis Straus, Nikolas Beck, Marko Frings, Sebastian Zensen, Rene Hosch, Giulia Baldini, Felix Nensa, Marcel Opitz, Johannes Haubold

**Affiliations:** 1 Institute of Diagnostic and Interventional Radiology and Neuroradiology, University Hospital Essen, Essen, Germany; 2 Institute for Artificial Intelligence in Medicine, University Hospital Essen, Essen, Germany; 3 Center for Interstitial and Rare Lung Diseases, Department of Pneumology, University Hospital Essen, Essen, Germany; 4 Department of Pneumology, University Hospital Essen, Essen, Germany

**Keywords:** Body composition, Lung diseases (interstitial), Lung volume measurements, Pulmonary disease (chronic obstructive), Tomography (x-ray computed)

## Abstract

**Background:**

Non-malignant chronic diseases remain a major public health concern. Given the alterations in lipid metabolism and deposition in the lung and its association with fibrotic interstitial lung disease (fILD) and chronic obstructive pulmonary disease (COPD), this study aimed to detect those alterations using computed tomography (CT)-based analysis of pulmonary fat attenuation volume (CTpfav).

**Methods:**

This observational retrospective single-center study involved 716 chest CT scans from three subcohorts: control (*n* = 279), COPD (*n* = 283), and fILD (*n* = 154). Fully automated quantification of CTpfav based on lung segmentation and HU-thresholding. The pulmonary fat index (PFI) was derived by normalizing CTpfav to the CT lung volume. Statistical analyses were conducted using Kruskal–Wallis with Dunn’s *post hoc* tests.

**Results:**

Patients with fILDs demonstrated a significant increase in CTpfav (median 71.0 mL, interquartile range [IQR] 59.7 mL, *p* < 0.001) and PFI (median 1.9%, IQR 2.4%, *p* < 0.001) when compared to the control group (CTpfav median 43.6 mL, IQR 16.94 mL; PFI median 0.9%, IQR 0.5%). In contrast, individuals with COPD exhibited significantly reduced CTpfav (median 36.2 mL, IQR 11.4 mL, *p* < 0.001) and PFI (median 0.5%, IQR 0.2%, *p* < 0.001).

**Conclusion:**

The study underscores the potential of CTpfav and PFI as imaging biomarkers for detecting changes in lung lipid metabolism and deposition and demonstrates a possibility of tracking these alterations in patients with COPD and ILDs. Further research is needed to validate these findings and explore the clinical relevance of CTpfav and PFI in lung disease management.

**Relevance statement:**

This study introduces a fully automated method for quantifying CTpfav, potentially establishing it as a new imaging biomarker for chronic lung diseases.

**Key Points:**

- This retrospective observational study employed an open-source, automated algorithm for the quantification of CT pulmonary fat attenuation volume (CTpfav).
- Patients with fibrotic interstitial lung disease (fILD) showed a significantly higher CTpfav and pulmonary fat index (PFI), *i.e*., CTpfav/CT lung volume, compared to a control group.
- Patients with chronic obstructive pulmonary disease (COPD) showed significantly lower CTpfav and PFI compared to the control group.
- CTpfav and PFI may each serve as imaging biomarkers for various lung diseases and warrant further investigation.

**Graphical Abstract:**

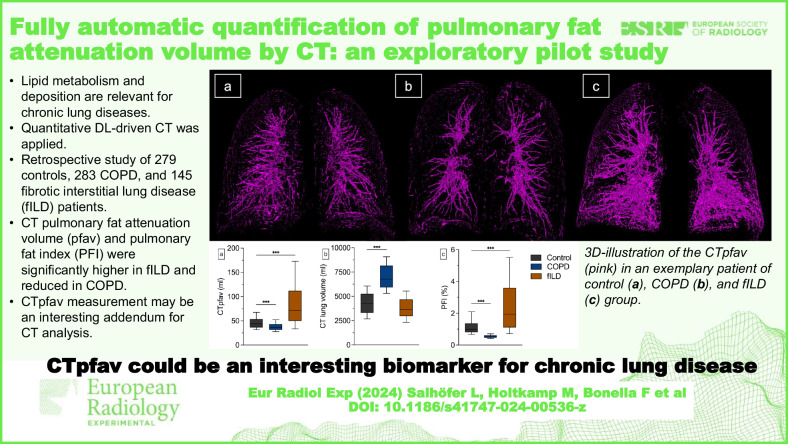

## Background

Lung diseases remain a significant public health concern in the 21st century, as non-malignant chronic respiratory disorders were the third leading cause of death in 2019, accounting for 4.0 million fatalities globally [1]. These compelling statistics underscore the critical significance of finding new biomarkers in the management of these conditions.

Even though the lung itself is not perceived as a fat-metabolizing organ for systemic circulation, it does have an active lipid metabolism to maintain its function [2]. Both clinically and scientifically, the surfactant is the best-understood pulmonary factor, consisting predominantly of lipids, as it is substantial in reducing the alveolar surface tension and preventing alveolar collapse. However, in recent years, there has been growing interest in the relationship between additional lipid pathways and different lung diseases. For instance, in chronic obstructive pulmonary disease (COPD), which is marked by the emphysematous destruction of alveoli and remodeling of airways, an alteration in the composition and amount of surfactant has been observed [3]. In recent years, multiple connections have been made between dysregulated lipid metabolism and the pathogenesis of lung fibrosis, covering profibrotic and antifibrotic aspects [4]. The vast majority of the studies focused on idiopathic pulmonary fibrosis (IPF), the most common and, at the same time, deadliest interstitial lung disease (ILD) [5, 6]. In patients with IPF and a fibrotic mice model, increased lipid deposition was observed in histopathological exams [7, 8]. Interestingly this observation preceded maximum fibrosis [7]. In 2022, Husseini et al [9] pioneered the histologic reporting of intraparenchymal adipocyte deposition in fibrotic lung diseases, including IPF and fibrotic non-specific interstitial pneumonia (fNSIP), further intensifying inquiries into its function in fibrogenesis. Although a connection between IPF and subpleural fat on CT scans has been established [10], O’Callaghan et al [11] were the first to demonstrate a total segmentation of pulmonary fat attenuation volume on CTs (CTpfav) with increased volumes in patients with IPF. Nevertheless, they utilized the manufacturer’s licensed platform for the semi-automatic measurements, which also required hands-on work [11].

Methods of cross-sectional imaging have already been successfully implemented and, in some cases, validated to characterize tissues [12, 13]. Additionally, quantitative CT imaging of the lung parenchyma has also been successfully established and linked to disease progression in chronic pulmonary conditions like cystic fibrosis or COPD [14, 15].

Advancements in machine learning have led to the development of new techniques for automatically extracting features from CT scans. This allows the investigation of specific biomarkers such as the volume of body components (*e.g*., subcutaneous adipose tissue) or organs (*e.g*., lung) [16–20]. These developments enable the assessment of biomarkers in both chronic and acute lung diseases [20–22] but, unlike their manual or semi-automatic predecessors, can also be seamlessly integrated into the clinical workflow [17]. Thus, following the pioneering work of O’Callaghan et al [11], the CTpfav could also be captured as a fully automated parameter at the point of care.

We hypothesize that by demonstrating a fully automated and open-source method for CTpfav quantification, we can further attempt to identify the already known alterations in lipid metabolism and deposition in lung tissue on CT scans. This might set the stage for subsequent studies aimed at potentially recognizing CTpfav as an imaging biomarker.

## Methods

### Ethics

This study was approved by the local institutional review board (approval number: 23-11410-BO). The institutional review board waived the requirement of written informed consent due to the retrospective nature of the study. Data underwent full anonymization before inclusion.

### Cohort definition and patient characteristics

**Fig. 1:**
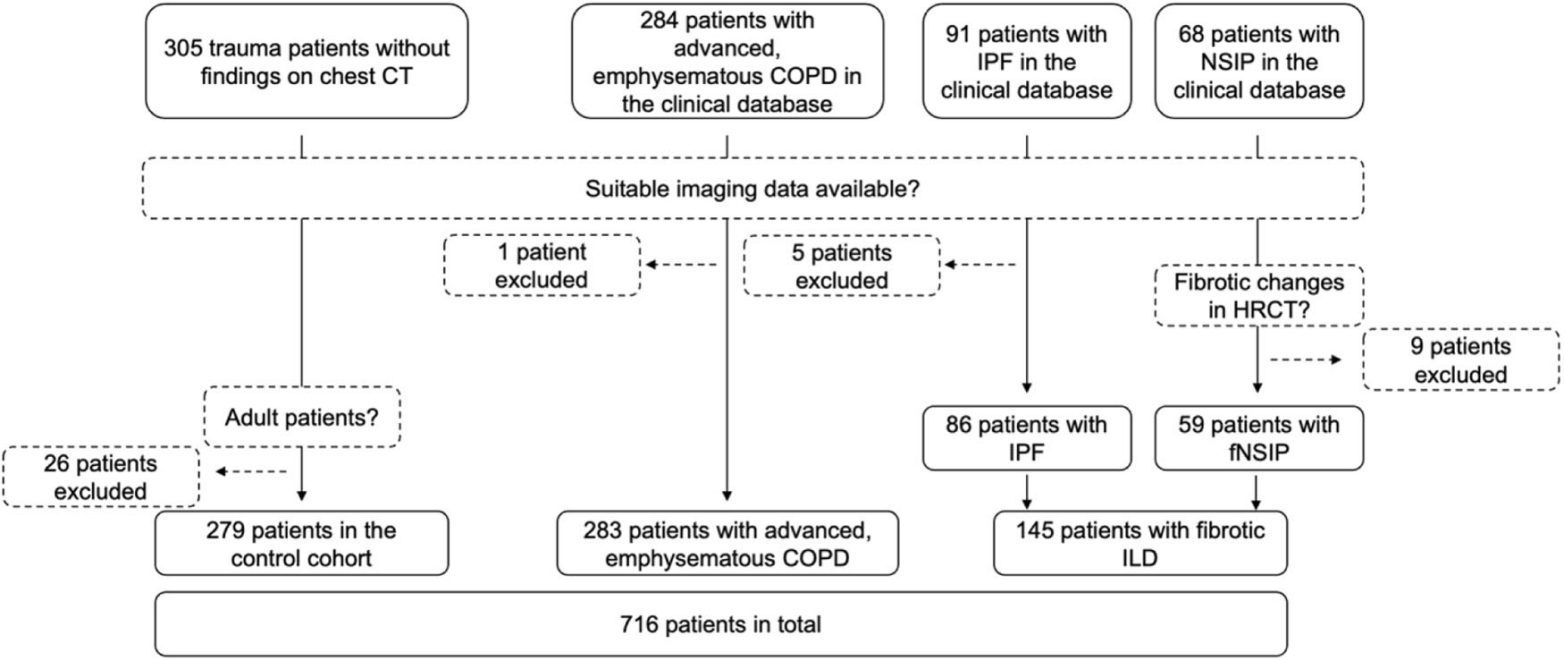
Depiction of the entire cohort and its subgroups. The investigated cohort of 716 patients comprised 279 patients in the control group, 283 patients with advanced COPD, and 145 patients with fILDs. COPD, Chronic obstructive pulmonary disease; fILD, Fibrotic interstitial lung disease; fNSIP, Fibrotic non-specific interstitial pneumonia; PFI Pulmonary fat index

For this retrospective observational study, the control cohort was created by including all trauma patients over the last three years (scans from January 2021 to September 2023) without pulmonary or cardiac comorbidities receiving CT imaging of the chest without any pathologic findings (*n* = 305). Twenty-six children were excluded resulting in 279 adult patients being included, with a median age of 41 years (interquartile range [IQR] 36 years), 104 of them being females (37%). Primarily, 284 patients with advanced, emphysematous COPD who underwent bronchoscopic volume reduction without inflammatory or malignant findings were included based on a local database. One patient was excluded, because of missing imaging data. Thus, the investigated COPD cohort comprised 283 patients with a median age of 63 years (IQR 11 years), 167 of them being females (59%). Furthermore, 145 patients with fibrotic ILDs (fILD) were included, with a median age of 68 years (IQR 16 years), 48 of them being females (33%), composed of 86 patients with IPF with a median age of 69 years (IQR 16 years), 18 of them being females (21%), diagnosed according to the ATS/ECR/JRS/ALAT guidelines [23], and 59 patients with fNSIP with a median age of 66 years (IQR 20 years), 30 of them being females (51%), based on an ILD-board decision. Primarily, 68 patients with non-specific interstitial pneumonia (NSIP) were considered for inclusion in the study, but nine patients without fibrotic changes were excluded based on a consensual decision of two experienced radiologists with 7 and 10 years of experience in lung CT (Fig. 1, Table 1). Before the study was conducted, five patients with IPF were excluded because of unsuitable imaging data.

**Table 1:** Demographic characteristics of the studied subjects

	Control (*n* = 279)	COPD (*n* = 283)	fILD (*n* = 145)
Age, years*	41 (26–62)	63 (58–69)	68 (59–75)
Sex, female	104 (37%)	167 (59%)	48 (33%)

* Data are medians with interquartile range

*COPD* Chronic obstructive pulmonary disease, *fILD* Fibrotic interstitial lung disease

### Extraction of the CTpfav and pulmonary fat index

Thin slice axial datasets of chest CT examinations, obtained on different scanners (SOMATOM Definition AS 64 or SOMATOM Definition Edge [Siemens Healthineers, Erlangen, Germany]), with a soft tissue kernel were loaded into the network. The lung segmentations were extracted using the TotalSegmentator within the Body and Organ Analysis (BOA) framework, which employs a variation of a nnU-Net [16, 17]. Within the lung segmentations, HU-thresholding of fat isodense voxels (from -200 to -60 HU) was utilized [11]. For normalization, CTpfav was divided by the CT lung volume to account for the differences in human and/or pulmonary size to obtain the pulmonary fat index (PFI), as follows:

$${Pulmonary\; fat\; index}=\frac{{CTpfav}[{mL}]}{{Lung\; volume}[{mL}]} \, \times \, 100$$

The fully automated extraction of the CTpfav is included in the BOA package as open-source software and is available via GitHub (https://github.com/UMEssen/Body-and-Organ-Analysis). For the subpleural subanalysis, distance measurements (1 cm) were performed by calculating a SignedMaurerDistanceMap using the SimpleITK (version 2.3.1) Python package, taking the image spacing into account to ensure accurate distance mapping.

Images were created using ITK-snap (version 4.0.2) with the following preferences for three-dimensional rendering: activated Gaussian image smoothing (standard deviation 0.50, maximum approximation error 0.03), activated Laplacian mesh smoothing (convergence 0.000, feature angle 45.0, iterations 50.0, relaxation 0.0100, deactivated feature edge smoothing, and deactivated boundary smoothing).

### Statistical analysis

The normality of the data distribution was evaluated using a D’Agostino and Pearson test [24]. Subsequently, given the non-normally distributed nature of the data, statistical significance was examined by a Kruskal–Wallis test. *Post hoc* analysis was conducted by Dunn’s multiple comparison test to assess intergroup differences.

The significance level was set at *p* = 0.05, and the analyses were conducted and displayed with GraphPad Prism (10.1.1) for MacOS. If not otherwise specified, data is given as median with IQR in brackets.

## Results

In a total of 716 chest CT scans from the control group and COPD/fILD cohorts, CTpfav, CT lung volume, and PFI were analyzed. In the control group, the median CTpfav was 43.6 mL (IQR 36.5–53.4 mL). CTpfav was significantly lower in patients with advanced COPD (36.2 mL, IQR 31.4–42.8 mL, *p* < 0.001). In patients with fILDs, the CTpfav demonstrated significantly higher median values with a larger IQR (median 71.5 mL, IQR 50.0–111.9 mL, *p* < 0.001) compared to the control group, respectively (Table 2).

**Table 2:** Results of CTpfav and CT lung volume segmentation, and PFI calculation across the different entities

	Control	COPD	fILD
CTpfav, mL	43.6 (36.5–53.4)	36.2 (31.4–42.8)	71.03 (50.0–111.9)
CT lung volume, mL	4,228 (3,312–5,259)	6,765 (5,922–8,155)	3,614 (2,959–4,680)
PFI, %	0.98 (0.80–1.35)	0.53 (0.46–0.61)	1.94 (1.11–3.60)

Data are medians with interquartile range

*COPD* Chronic obstructive pulmonary disease, *CTpfav* Computed tomography pulmonary fat attenuation volume, *fILD* Fibrotic interstitial lung disease, *PFI* Pulmonary fat index

**Fig. 2:**
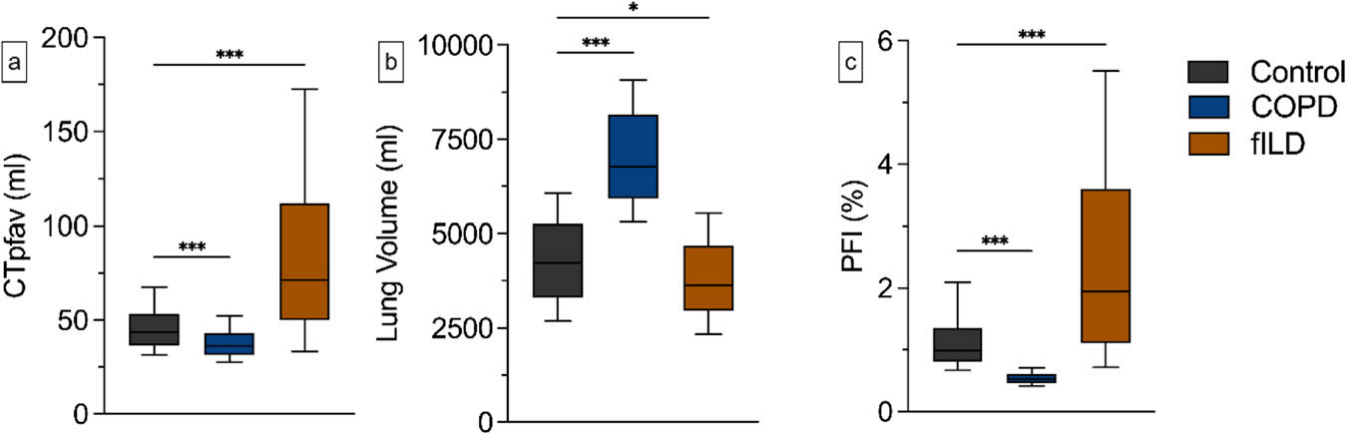
Comparison of the PFI among the different entities. Advanced COPD patients exhibited a significantly lower median CTpfav (36.2 mL, IQR 36.5–53.4 mL, *p* < 0.001 (**a**)), while patients with fILDs showed a significantly higher median CTpfav (71.5 mL, IQR 50.0–111.9 mL, *p* < 0.001 (**a**)). Compared to the control group, COPD patients had a significantly higher CT lung volume (6,765 mL, IQR 5,922–8,155 mL, *p* < 0.001 (**b**)). Conversely, patients with fILDs displayed a lower CT lung volume (3,626 mL, IQR 2,959–4,680 mL, *p* = 0.012 (**b**)). The PFI was significantly higher in patients with fILDs (2.0%, IQR 1.1–3.6%, *p* < 0.001 (**c**)). Conversely, a significantly lower PFI was observed in COPD (0.5%, IQR 0.5–0.6%, *p* < 0.001 (**c**)). Whiskers represent the 10th and 90th percentile. Only statistical significance compared to control is reported. *p < 0.05, ***p < 0.001. CTpfav, Computed tomography pulmonary fat attenuation volume; COPD, Chronic obstructive pulmonary disease; fILD, Fibrotic interstitial lung disease; PFI, Pulmonary fat index

**Fig. 3:**
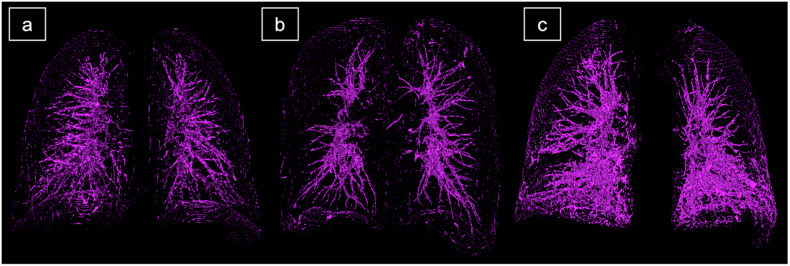
Exemplary 3D presentation of the CTpfav for one patient of each group. 3D-representation of the CTpfav for the different groups: **a** Control (42.1 mL), (**b**) COPD (36.1 mL), and (**c**) fILD (74.4 mL). 3D, Three-dimensional; CTpfav, Computed tomography pulmonary fat attenuation volume; COPD, Chronic obstructive pulmonary disease; fILD, Fibrotic interstitial lung disease

Regarding the CT lung volume, significant differences were observed in both investigated groups compared to the control. Patients with advanced COPD had a significantly higher CT median lung volume (6,765 mL, IQR 5,922–8,155 mL, *p* < 0.001), whereas patients with an fILD exhibited a lower median CT lung volume (3,626 mL, IQR 2,959–4,680 mL, *p* = 0.012). In the control group, the median PFI was 0.98% (IQR 0.80–1.35%). Compared to the control, patients with fILDs exhibited a significantly higher median PFI (1.94%, IQR 1.11–3.60%, *p* < 0.001), while a lower median PFI was observed in COPD patients (0.53%, IQR 0.46–0.61%, *p* < 0.001) (Figs. 2 and 3).

**Fig. 4:**
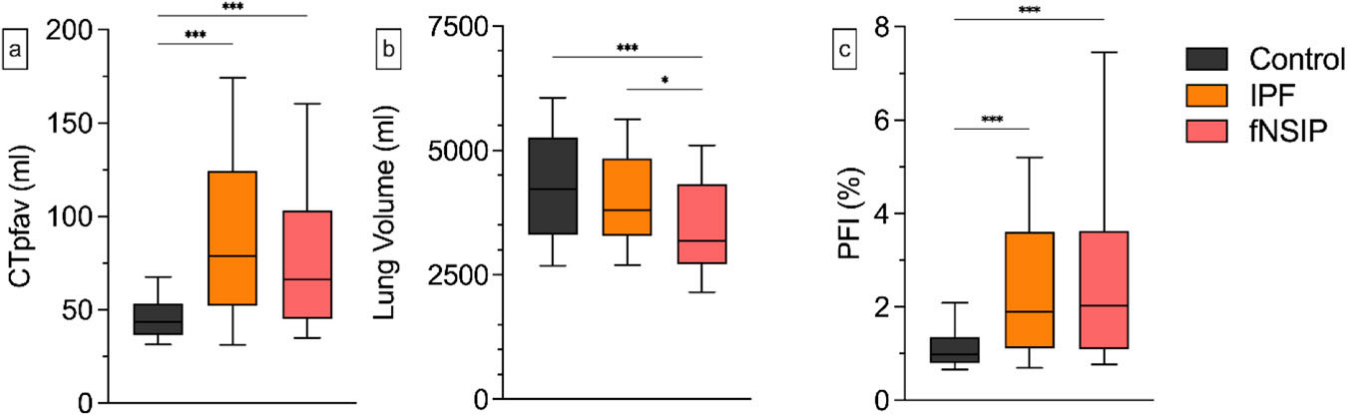
Subanalysis of the PFI among fILD patients. Patients with IPF (CTpfav 78.9 mL, IQR 52.1–124.4 mL, *p* < 0.001; PFI 1.9%, IQR 1.1–3.6%, *p* < 0.001 (**a** + **c**)), and fNSIP (CTpfav 66.1 mL, IQR 45.1–103.2 mL, *p* < 0.001; PFI 2.0%, IQR 1.1–3.6%, *p* < 0.001 (**a** + **c**)) had significantly higher CTpfav and PFI compared to the control group (CTpfav 43.6 mL, IQR 36.5–53.4 mL, 1.0%, IQR 0.8–1.4% (**a** + **c**) but did not show significant differences among each other. Concerning the CT lung volume, patients with fNSIP (3,178 mL, IQR 2,959–4,680 mL (**b**)) demonstrated significantly lower values compared to the IPF (3,803 mL, IQR 3,282–4,839 mL, *p* = 0.016 (**b**)) and control group (4,228 mL, IQR 3,312–5,259 mL, *p* < 0.001 (**b**)). Whiskers represent the 10th and 90th percentile. *p < 0.05, ***p < 0.001. CTpfav, Computed tomography pulmonary fat attenuation volume; COPD, Chronic obstructive pulmonary disease; fILD, Fibrotic interstitial lung disease; fNSIP, Fibrotic non-specific interstitial pneumonia; PFI, Pulmonary fat index

In the subanalysis of the fILD group, patients with IPF and fNSIP exhibited significantly higher median CTpfav (IPF 78.98 mL, IQR 52.1–124.4 mL, *p* < 0.001; fNSIP 66.1 mL, IQR 45.1–103.2 mL, *p* < 0.001) and PFI (IPF 1.9%, IQR 1.1–3.6%, *p* < 0.001; fNSIP PFI 2.0%, IQR 1.1–3.6%, *p* < 0.001) than the control group. Additionally, only patients with fNSIP demonstrated a significantly reduced CT lung volume relative to the control group (3,178 mL, IQR 2,717–4,325 mL, *p* < 0.001). The only significant difference between the IPF and fNSIP groups was the CT lung volume, with the fNSIP group having smaller volumes (IPF 3,803 mL, IQR 3,282–4,839 mL, *p* = 0.016). Furthermore, patients with IPF displayed a tendency towards higher CTpfav compared to the fNSIP group (Fig. 4).

Visually (see Fig. 3), there is an accentuation of CTpfav around the vascular systems and in the subpleural space. Therefore, a subanalysis of the subpleural and nonsubpleural PFI was conducted to unveil further differences among the different entities. Patients with fILD had the highest median subpleural PFI (3.7%, IQR 2.3–6.6%) with significant elevation compared to the nonsubpleural PFI in fILD (1.2%, IQR 0.7–2.1%, *p* < 0.001) and the subpleural zone in the control group (1.5%, IQR 1.3–2.3%, *p* < 0.001) and COPD group (1.0%, IQR 0.9–1.1%, *p* < 0.001). Compared to the subpleural zone, there was a significantly lower value within the nonsubpleural zone for the control group (0.79%, IQR 0.64–1.03%, *p* < 0.001) and COPD group as well (0.41%. IQR 0.36–0.48%, *p* < 0.001). Additionally, all subpleural and nonsubpleural assessments significantly varied between the different diseases (*e.g*., COPD nonsubpleural *versus* control nonsubpleural, *p* < 0.001) (Supplementary material S1).

Analog to the analysis of the total lung, there were significant differences between the different entities concerning the lobar subanalysis (*e.g*., controls lower lobes *versus* fILD lower lobes: 0.9%, IQR 0.7–1.1% *versus* 3.0%, IQR 1.7–5.2%, *p* < 0.001). In the fILD group, the lower lobes exhibited the highest PFI values, with significant differences compared to the middle lobe (1.4%, IQR 0.9–3.0%, *p* < 0.001) and the upper lobes, which had the lowest PFI (1.2%, IQR 0.7–2.4%, *p* < 0.001). In the COPD group, the lowest PFI was observed in the upper lobes (0.5%, IQR 0.4–0.6%), with significantly higher values in the lower lobes (0.6%, IQR 0.5–0.7%, *p* = 0.01) and a tendency to higher values in the middle lobe (0.8%, IQR 0.6–0.9). Significant differences were also observed between the individual lobes in the control group, with the lower lobes exhibiting the highest PFI values (1.1%, IQR 0.9–1.8%) (Supplementary material S2).

## Discussion

This study aimed to establish a fully automated and open-source method for CTpfav quantification to facilitate the identification of alterations in lipid metabolism and deposition in lung tissue and to establish a new imaging biomarker for patients with chronic lung disease from CT scans. CTpfav measurements were normalized to the CT lung volume to account for patient size, resulting in the creation of the PFI. We identified a significant elevation in patients with fILD and a reduction in patients with COPD, compared to a control, respectively.

The finding that patients with ILDs had a significantly higher PFI than the controls underscores the recent results of O’Callaghan et al in their study of patients with IPF [11]. While changes in lipid metabolism have been linked to profibrotic and antifibrotic processes in ILDs multiple times [4], there are fewer studies correlating fat deposition in lung parenchyma with fibrosis in IPF/ILD [7, 8]. In 2022, Husseini et al [9] were the first to demonstrate parenchymal lipid deposition over a wider range of fibrotic lung diseases, including NSIP, emphasizing its impact on fibrogenesis. Various studies have shown that imaging biomarkers from CT play a role in estimating the prognosis of patients with IPF, *e.g*., linking a lower muscle volume or a smaller area at certain levels on CT with reduced survival [25, 26]. Densitometry and histogram-based analyses of the lung have already proven to be possible parameters for estimating pulmonary function and also for estimating transplant-free survival [27, 28].

Our investigation visualizes CTpfav primarily around the vascular system and in the subpleural space, being more pronounced in the ILD group than in the controls. Therefore, our study illustrates a CT correlation for the histologically recognized subpleural fat deposits in lung fibrosis [9] as we observed a significantly elevated subpleural PFI in the fILD group, both in comparison to the nonsubpleural lung regions within the fILD group and to the subpleural spaces in the control and COPD groups. Additionally, fILD patients had a significant lower lobe accentuation of CTpfav corresponding to the clinically observed predilection in fibrotic lung abnormalities such as fNSIP and IPF [29]. Those two findings foster the PFI as a possible surrogate marker for the course of fILDs. Otherwise, there is visually more pronounced CTpfav around the pulmonary vascular system. The quantity of vessel-related structures in CT imaging has been reported as a predictor for survival and lung function decline by Chung et al [30]. Hence, it is particularly interesting to further analyze the perivascular CTpfav, as it may represent an increased amount of local adipose tissue. Nonetheless, it is likely that a partial volume effect at the transition from vascular structures to aerated lung tissue accounts for at least part of this phenomenon. Increased lung vascularity in patients with ILD may further amplify this effect [31].

Furthermore, the data spread for the CTpfav in the fILD group is rather large (median: 71.5 mL, IQR 50.0–111.9 mL) compared to the control and COPD groups. This is likely due to the heterogeneity of the disease course or stage, respectively, making it difficult to distinguish between ILD and a healthy lung based on a single CTpfav value. The phenomenon that individual values cannot always be accurately categorized within established reference ranges is particularly well-known in the form of false-positive test results in other areas [32]. To reduce the susceptibility to false-positive or negative findings, large multi-centric studies with a diverse patient collective are needed. Additionally, once the relationship between CTpfav and disease severity is better understood, the trend of CTpfav will likely become more interesting than a single value, potentially serving as a valuable parameter for disease monitoring.

A different picture emerges in patients with COPD, as the CT-based quantification shows significantly increased CT lung volume along with lower CTpfav and PFI compared to the control group. The combination of dramatically increased CT lung volume and decreased CTpfav results in a significantly lower PFI. Since the emphysematous destruction of lung tissue is a key feature of progressing COPD, it naturally leads to an increased CT lung volume. Pulmonary lipid metabolism is altered in multiple manners in patients with COPD [33]. Nevertheless, evidence regarding the lipid content within the pulmonary tissue is scarce. Agudelo et al [3] observed a reduction of the surfactant volume combined with a changed composition. The decreased CTpfav in our study might be the first hint in cross-sectional imaging that supports this thesis of reduced pulmonary fat volume in COPD. Despite that, the extent of pulmonary vascularization may also play a role concerning CTpfav. As parts of CTpfav are distributed perivascular and COPD is associated with lower vascular volume [34], this might contribute to the lower CTpfav and subsequently PFI. Upper lobe predilection is a common feature in emphysematous COPD. Appropriately, PFI in the upper lobes of the COPD group is significantly lower compared to both the upper lobes of the Control and fILD groups, as well as the lower lobes of the COPD group.

This exploratory study has to address several limitations. Although a large number of patients and CT scans were included, the presented study is still limited by its monocentric design. The HU range for the CTpfav was set at values from -200 to -40, aligning with those used in the study by O’Callaghan et al [11]. Here, validation, for example, through a phantom study, could be meaningful. A possible limitation of the CTpfav is the partial volume effect, which can occur during the transition from soft tissue to ventilated lung. Thus, the emphasis of the CTpfav around vessels already visible in the control could partly be attributed to this. Nonetheless, it might also represent perivascular adipose tissue, which could be understood as a sign of angiogenesis due to increased vascularization in patients with IPF/ILDs [31]. For IPF, a connection between CTpfav and lung function, as well as other disease features, has already been established [11]. With the applied technique, endobronchial mucus may also be annotated. Although we demonstrated significant differences between subpleural and nonsubpleural PFI, future studies should investigate CTpfav in even more compartments, such as peribronchial and perivascular. Lobar subanalysis of the PFI is an interesting feature per se, but incomplete interlobar fissures are a common normal variant. This might lead to inaccurate segmentations of all pulmonary lobes, of which the middle lobe is most prone to error as it comes with the lowest volume. Additionally, a combination of the BOA framework with the CTpfav volumetry and already existing methodologies for fat quantification would be desirable. For instance, dual-energy CT enables contrast agent-independent fat quantification [35, 36]. As Morsbach et al [37] raised concerns about the methodological inaccuracy in planimetric BCA at different energy levels, validation studies are urgently needed to assess the accuracy of volumetric BCA. Studies of this kind would also be desirable for other entities to better assess its clinical relevance. With the open-source publication of our fully automated algorithm in the BOA framework, we want to facilitate this.

Finally, it seems to be feasible to track changes in lipid metabolism and deposition known from histopathology and pathophysiology in CT images using CTpfav. Adjusted for CT lung volume, the PFI might be an interesting imaging biomarker to investigate in the future. Although significant differences were observed in patients with ILD and COPD, further investigations of the PFI are urgently needed to comprehensively understand its clinical relevance.

## Supplementary information

**Additional file 1: Supplementary Material 1:** Subanalysis of the PFI in the subpleural and nonsubpleural areas across the different entities. Patients with fILD had significantly higher subpleural PFI than nonsubpleural PFI (3.73%, IQR 2.83–6.60% vs. 1.21%, IQR 0.68–2.05%, *p* < 0.001). Moreover, subpleural PFI in fILD was markedly higher compared to COPD (1.01%, IQR 0.89–1.14%, *p* < 0.001) and the control group (1.53%, IQR 1.26–2.32%, *p* < 0.001). Additionally, both the control and COPD groups showed significantly higher subpleural PFI values (control: 1.53%, IQR 1.26–2.32 IQR) vs. 0.79%, IQR 0.64–1.03%, *p* < 0.001; COPD: 1.00%, IQR 0.89–1.14% vs. 0.41%, 0.36–0.48% and significant differences in regional PFI between the entities. Whiskers represent the 10th and 90th percentile. ***p < 0.001. **Supplementary Material 2:** Subanalysis of the PFI in the different lobes across the different entities. Significant differences in the PFI were observed between the individual lobes across the different entities. For instance, patients with COPD had the lowest PFI in the upper lobe (0.49%, IQR 0.41–0.60%) compared to patients in the control group (0.86%, IQR 0.71–1.11%, *p* < 0.001) and the ILD group (1.19%, 0.68–2.35%, *p* < 0.011). Additionally, there were intra-entity differences between the lobes. Patients in the ILD group showed a significantly higher PFI in the lower lobe (2.95%, IQR 1.69–5.20%) compared to both the upper and middle lobes (upper lobe: *p* < 0.011; middle lobe: 1.42%, IQR 0.94–3.05%, *p* < 0.001). Similarly, patients in the COPD group had the lowest PFI in the upper lobe, with a significant difference compared to the lower lobe (0.56%, IQR 0.46–0.68%, *p* = 0.01). Whiskers represent the 10th and 90th percentile. *p < 0.05, ***p < 0.001.

## Data Availability

The datasets used and/or analyzed during the current study are available from the corresponding author upon reasonable request.

## Abbreviations

BOA: Body and organ analysis
COPD: Chronic obstructive pulmonary disease
CT: Computed tomography
CTpfav: CT pulmonary fat attenuation volume
fILD: Fibrotic interstitial lung disease
fNSIP: Fibrotic non-specific interstitial pneumonia
ILD: Interstitial lung disease
IPF: Idiopathic pulmonary fibrosis
IQR: Interquartile range
NSIP: Non-specific interstitial pneumonia
PFI: Pulmonary fat index
