# Supplementary material for: Fully automatic quantification of pulmonary fat attenuation volume by CT: an exploratory pilot study
Source: Eur Radiol Exp. 2024 Dec 5;8:139. doi: 10.1186/s41747-024-00536-z (PMC11621257; doi:10.1186/s41747-024-00536-z)
Supplement: Supplementary file 1 — Additional file 1: Supplementary Material 1: Subanalysis of the PFI in the subpleural and nonsubpleural areas across the different entities. Patients with fILD had significantly higher subpleural PFI than nonsubpleural PFI (3.73%, IQR 2.83–6.60% vs. 1.21%, IQR 0.68–2.05%, p < 0.001). Moreover, subpleural PFI in fILD was markedly higher compared to COPD (1.01%, IQR 0.89–1.14%, p < 0.001) and the control group (1.53%, IQR 1.26–2.32%, p < 0.001). Additionally, both the control and COPD groups showed significantly higher subpleural PFI values (control: 1.53%, IQR 1.26–2.32 IQR) vs. 0.79%, IQR 0.64–1.03%, p < 0.001; COPD: 1.00%, IQR 0.89–1.14% vs. 0.41%, 0.36–0.48% and significant differences in regional PFI between the entities. Whiskers represent the 10th and 90th percentile. ***p < 0.001. Supplementary Material 2: Subanalysis of the PFI in the different lobes across the different entities. Significant differences in the PFI were observed between the individual lobes across the different entities. For instance, patients with COPD had the lowest PFI in the upper lobe (0.49%, IQR 0.41–0.60%) compared to patients in the control group (0.86%, IQR 0.71–1.11%, p < 0.001) and the ILD group (1.19%, 0.68–2.35%, p < 0.011). Additionally, there were intra-entity differences between the lobes. Patients in the ILD group showed a significantly higher PFI in the lower lobe (2.95%, IQR 1.69–5.20%) compared to both the upper and middle lobes (upper lobe: p < 0.011; middle lobe: 1.42%, IQR 0.94–3.05%, p < 0.001). Similarly, patients in the COPD group had the lowest PFI in the upper lobe, with a significant difference compared to the lower lobe (0.56%, IQR 0.46–0.68%, p = 0.01). Whiskers represent the 10th and 90th percentile. *p < 0.05, ***p < 0.001. [file 41747_2024_536_MOESM1_ESM.pdf]

Fully automatic quantification of pulmonary fat attenuation volume by CT: an exploratory pilot study

ELECTRONIC SUPPLEMENTARY MATERIAL

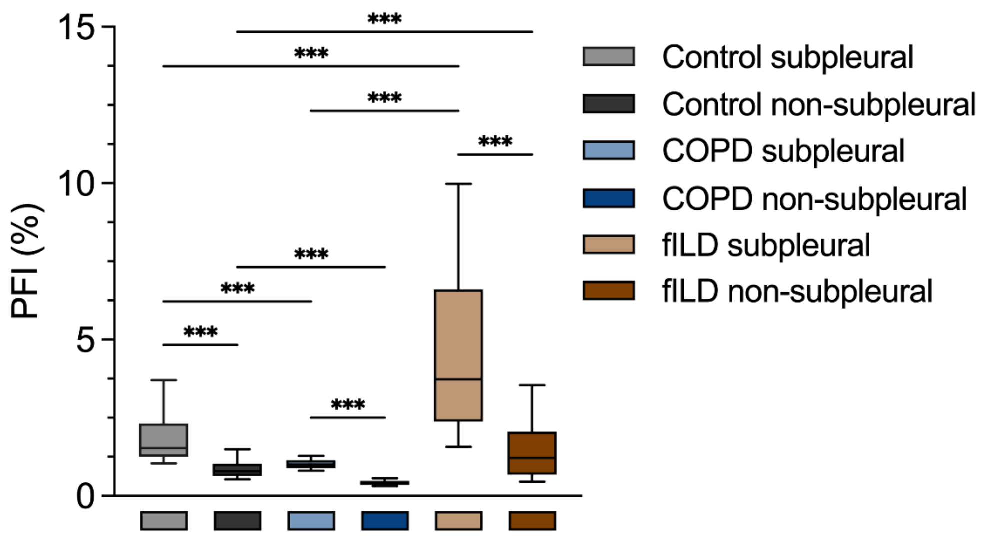

Supplementary Material 1: Subanalysis of the PFI in the subpleural and non-subpleural areas across the different entities. Patients with fILD had significantly higher subpleural PFI than non-subpleural PFI (3.73% , IQR 2.83–6.60 % vs. 1.21 %, IQR 0.68-2.05 %, p < 0.001). Moreover, subpleural PFI in fILD was markedly higher compared to COPD (1.01%, IQR 0.89–1.14 %, p < 0.001) and the control group (1.53 %, IQR 1.26–2.32 %), p < 0.001). Additionally, both the control and COPD groups showed significantly higher subpleural PFI values (control: 1.53 %, IQR 1.26 – 2.32 IQR) vs. 0.79 %, IQR 0.64 –1.03 %, *p* < 0.001; COPD: 1.00%, IQR 0.89 – 1.14 % vs. 0.41 %, 0.36 – 0.48 %) and significant differences in regional PFI between the entities. Whiskers represent the 10th and 90th percentile. *** = *p* < 0.001. *COPD* Chronic obstructive pulmonary disease, *fILD* Fibrotic interstitial lung disease, *PFI* Pulmonary fat index.

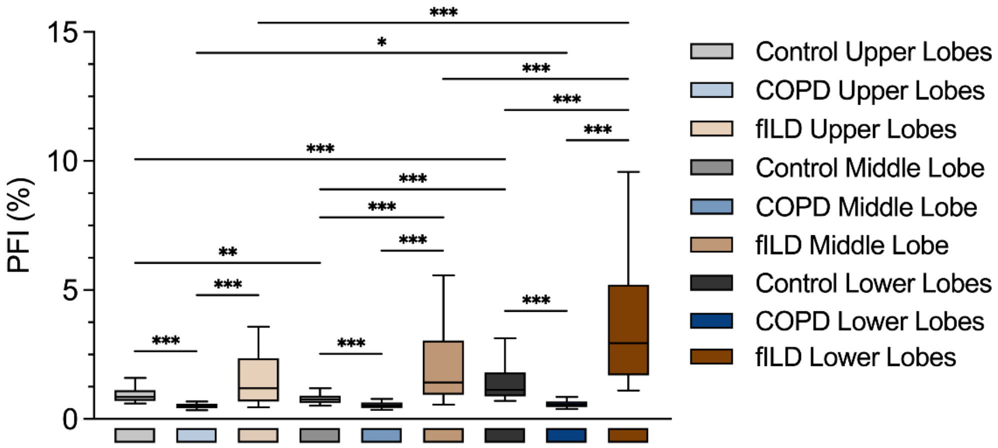

Supplementary Material 2: Subanalysis of the PFI in the different lobes across the different entities. Significant differences in the PFI were observed between the individual lobes across the different entities. For instance, patients with COPD had the lowest PFI in the upper lobe (0.49 %, IQR 0.41 – 0.60 %) compared to patients in the control group (0.86 %, IQR 0.71–1.11 %, $p < 0.001$) and the ILD group (1.19 %, 0.68 – 2.35 %, $p < 0.011$). Additionally, there were intra-entity differences between the lobes. Patients in the ILD group showed a significantly higher PFI in the lower lobe (2.95 %, IQR 1.69 – 5.20 %) compared to both the upper and middle lobes (upper lobe: $p < 0.011$; middle lobe: 1.42 %, IQR 0.94–3.05 %, $p < 0.001$). Similarly, patients in the COPD group had the lowest PFI in the upper lobe, with a significant difference compared to the lower lobe (0.56 %, IQR 0.46 – 0.68 %, $p = 0.01$). Whiskers represent the 10th and 90th percentile. * = $p < 0.05$, *** = $p < 0.001$ *COPD* Chronic obstructive pulmonary disease, *fILD* Fibrotic interstitial lung disease, *PFI* Pulmonary fat index.
